# Editorial: Probiotics and its Effects on Inflammatory and Infectious Disorders

**DOI:** 10.3389/fphar.2022.868044

**Published:** 2022-03-10

**Authors:** Siomar de Castro Soares, Helioswilton Sales-Campos

**Affiliations:** ^1^ Federal University of Triângulo Mineiro, Uberaba, Brazil; ^2^ Institute of Tropical Pathology and Public Health, Federal University of Goiás, Goiânia, Brazil

**Keywords:** inflammation, probiotics, infection, treatment, gut microbiota

## Introduction

Human beings have evolved surrounded by a variety of microorganisms. Aside from their presence as outer neighbors, bacteria, viruses, fungi, archaea and parasites, may also live in close proximity inhabiting distinct ecological niches in our body, and are known as microbiota (Simon et al., 2019). For decades, these “foreigners” were believed to have only very specific and limited roles towards the functionality of human biological systems. However, especially with the use of next generation sequencing (NGS), mainly targeting the largest population of living microorganisms inhabiting different body sites, which are bacteria, our knowledge in this field has drastically increased (Toju et al., 2020).

Currently, the microbiota of different body sites is believed to influence (or to be influenced by) distinct biological systems, including nervous, immune and endocrine, among others, thus directly contributing to the maintenance of healthy and disease states. Among these different microbial niches, the gut is by far, the richest (in diversity and composition) and the most explored in different contexts (Chen et al., 2018). However, it is not clear yet if microbial disturbances (known as dysbiosis) are a consequence or the causative agent of inflammatory and infectious diseases. Despite the ongoing advances in exploring microbial communities in the aforementioned scenarios, the therapeutic potential of microorganisms have been explored for centuries in human culture. Either as fermented foods (with unknown amounts and composition of microorganisms) or using known species of microbes at specific concentrations, such approach aims at modulating microbial composition and diversity, mainly gut microbiota, thus reestablishing microbial balance and constraining inflammation. Although some beneficial properties of probiotics have been explored in different contexts (Sales-Campos et al., 2019), the ongoing amount of research identifying the therapeutic potential of known/unknown microorganisms, used alone or in combination with classical therapies, may represent a new frontier in the field of microbiota manipulation, thus leading to a more favorable outcome of infectious and inflammatory diseases.

## Probiotics in Inflammatory Disorders

The mutual contribution between microbiota, mainly from Gastrointestinal tract (GIT), and different biological systems dictates the outcome of health and disease. In this view, Curciarello et al. investigate the potential anti-inflammatory effect of *Lactobacillus kefiri* in patients with Inflammatory Bowel Disease (IBD). More specifically, *L. kefiri* reduced the release of IL-6 and IL-8 from inflamed biopsies. Also, for the first time, they have reported the immunomodulatory effect of a kefir-isolated strain, *L. kefiri* CIDCA 8,348, on human intestinal tissue and primary T cells from IBD patients. Additionally, Belo et al. investigated the potential role of surface-layer proteins (Slp), notably SlpB from *Propionibacterium freudenreichii* CIRM-BIA 129*,* as a modulator of inflammation in Ulcerative colitis (UC). Mice exposed to DSS and treated with the probiotic *Lactococcus lactis* NCDO 2118 expressing a recombinant SlpB, had reduced severity of colitis and improved disease score, when compared to untreated mice. Also, it constrained inflammation in diseased mice. Finally, Savassi et al. developed a lyophilized synbiotic, to address its effects as adjuvant treatment in mucositis. The formulation reduced weight loss, intestinal permeability, and the intensity of inflammation in the duodenum, ileum, and colon; besides, it decreased the levels of pro-inflammatory cytokines. These data suggest probiotic bacteria as promising candidates for the treatment and prevention of GIT inflammatory diseases.

Strategies aiming at modulating the microbiota-gut-brain axis may represent a new frontier in developing therapeutic approaches for neuropsychiatric disorders. Wang et al. explored the role of the psychobiotic *Lactobacillus johnsonii* BS15 on the gut-brain axis to elucidate whether it could modulate the gut environment, thus, preventing memory dysfunction in an experimental model of psychological stress. The psychobiotic not only enhanced the performance of mice under stressing conditions, but also positively modulated the hypothalamic–pituitary–adrenal axis and memory-related functional proteins, besides maintaining gut barrier integrity.

In general, autoimmune or immune-mediated diseases are multifactorial disorders in which genetic mutations, environmental factors, immune imbalance and microbiota dysbiosis contribute to disease onset. Guo et al., reviewed the beneficial effects of probiotics in experimental models of Systemic Lupus Erythematosus (SLE) highlighting its impact towards a reduction in cardiovascular and renal complications. If the number of human studies addressing the role of probiotics in SLE is limited, Ferro et al., showed a completely different scenario for rheumatoid arthritis (RA). The probiotic bacteria *Lactobacillus casei* seems to represent the best candidate for application as adjuvant therapy for RA patients. In addition, Pagnini et al., discussed the potential role of probiotics on modulating the vitamin D pathway to treat IBD.

Obesity is marked by a low grade chronic inflammation and dysbiosis, and the review submitted by Maioli et al., explores the role of *Faecalibacterium prausnitzii* as a potential treatment and a putative biomarker in this scenario.

Liver inflammation, as a consequence of alcohol consumption, can evolve to more severe forms of liver damage, including cirrhosis and hepatocarcinoma, where such disturbances can be generically described as alcoholic liver disease (ALD). Also, ALD outcome is drastically influenced by gut dysbiosis which suggests a role for probiotics, in combination with classic approaches, to attenuate liver inflammation and ameliorate disease progression, as reviewed by Fuenzalida et al.

## Probiotics in Infectious Disorders

Members of the genus Bifidobacterium are the first to colonize the human gut, exerting health benefits for the host, and are also ubiquitously used as probiotics. Shimabukuro et al. evaluated the effect of two Bifidobacterium strains in inhibiting *Porphyromonas gingivalis* interaction with host cells and biofilm formation in periodontitis. More specifically, *Bifidobacterium bifidum* 1622A showed greater potential to control periodontitis, once it has not changed the inflammatory parameters significantly and prevented alveolar bone loss.

Cell-free supernatants of probiotic bacteria have been proposed lately as a safer option when compared to the use of live bacteria. Dubey et al. explored the use of cell-free supernatant of *Lactiplantibacillus plantarum* MTCC 2621 (Lp2621) to evaluate the potential antibacterial, hemolytic, antioxidant and wound healing properties using *in vitro* and *in vivo* approaches. Treatment with Lp2621 gel upregulated IL-6 in the early phase of wound healing and enhanced IL-10 expression in the later phase. Also, this treatment improved angiogenesis, proliferation of fibroblasts, re-epithelization, and recruitment of polymorphonuclear leukocytes.

Probiotics have also been used to treat other infectious disorders. The crosstalk between gut and lungs has been proposed as a key driver for host homeostasis. For this reason, gut microbiota dysbiosis also impacts lung function, thus increasing the susceptibility of respiratory tract infections. In this regard, Cruz et al. reviewed the role of prebiotics, probiotics and synbiotics on the prevention or as therapeutic approaches to treat bacterial, viral, fungal and helminthic infections affecting lungs. In this context, and based on the limitations concerning anthelmintic drugs, Saracino et al., highlighted the effects of probiotics to elicit a type 2 immune response, and therefore, improving the response and control against helminthic infection.

## Conclusion

During the past years, a growing number of researchers have been dedicating time and efforts to explore the role of probiotics in different scenarios. Likewise, the popularization of NGS drastically improved our knowledge about microbiota composition and ecology, which not only facilitated the identification of microorganisms with probiotic potential but also the improvement of already known species. Despite this promising scenario, the use of probiotics, especially in clinical practice, remains strict to only a few inflammatory diseases, mainly those affecting GIT. Unfortunately, the number of human studies addressing the role of probiotics in infectious diseases is even smaller. However, the rising number of experimental studies addressing the beneficial impact of probiotics, mainly as adjuvant therapy, and the association between its use and the reestablishment of gut microbial balance, may encourage a broader use by clinicians in the aforementioned scenarios.
